# Cross-cultural adaptation and validation of the Chinese version of the 25-question Geriatric Locomotive Function Scale in patients undergoing total knee arthroplasty

**DOI:** 10.3389/fmed.2025.1613589

**Published:** 2025-07-23

**Authors:** Zhipeng Tai, Dongping Wan, Shuxin Yao, Shaohua Miao, Qiang Zan, Yanchen Tan, Jianbing Ma, Chao Xu

**Affiliations:** ^1^Department of Knee Joint Surgery, Honghui Hospital, Xi’an Jiaotong University, Xi’an, China; ^2^The First Clinical Medical College, Shaanxi University of Chinese Medicine, Xianyang, China; ^3^The First Clinical Medical College, Guangxi University of Chinese Medicine, Nanning, China; ^4^Department of Orthopedics, Dali County Hospital, Weinan, China; ^5^Student Brigade of Basic Medical College, Air Force Military Medical University, Xi’an, China

**Keywords:** Geriatric Locomotive Function Scale, cross-cultural adaptation, total knee arthroplasty, knee osteoarthritis, Psychometrics, Chinese

## Abstract

**Background:**

Knee osteoarthritis (KOA) is prevalent among the elderly, often necessitating total knee arthroplasty (TKA) for severe cases. However, traditional assessment tools primarily focus on pain and physical function, neglecting the psychosocial aspects that influence postoperative satisfaction. The 25-question Geriatric Locomotive Function Scale (GLFS-25) is a comprehensive measure of locomotor function, however, it has limited validation within populations in Mainland China.

**Objectives:**

This study aimed to translate, cross-culturally adapt, and validate the Chinese version of the GLFS-25 (GLFS-25CV) for evaluating postoperative outcomes in TKA patients with KOA.

**Methods:**

Following established guidelines, the English GLFS-25 was both forward- and back-translated, subjected to expert review, and pretested in 30 patients. End-stage KOA patients scheduled for primary unilateral TKA were then recruited, with a subset completing the GLFS-25CV twice, one week apart, to assess test-retest reliability. Exploratory factor analysis (EFA) was conducted to evaluate structural validity, while convergent validity was assessed through Pearson correlations with the Oxford Knee Score (OKS), the Western Ontario and McMaster Universities Osteoarthritis Index (WOMAC), and the 5-Level EuroQol Five-Dimensional Questionnaire (EQ-5D-5L). Internal consistency was evaluated using Cronbach’s α, and standard deviations (SD) were calculated. Additionally, floor and ceiling effects were analyzed based on score distributions.

**Results:**

A total of 295 participants completed the study. Both the four-factor model identified by EFA, which explained 58% of the total variance, and the strong correlations with the OKS (rho = 0.85), the WOMAC (rho = 0.77), and the EQ-5D-5L (rho = 0.66) collectively confirm the excellent validity of the GLFS-25CV. Internal consistency was excellent (Cronbach’s α = 0.94, SD > 0.85), and test-retest reliability (ICC = 0.94–0.97) was also strong, confirming its overall reliability. Neither floor nor ceiling effects were significant, and no participants reported difficulty completing the instrument.

**Conclusion:**

The GLFS-25CV is a reliable, valid, and user-friendly tool for assessing knee function in individuals in mainland China undergoing TKA for KOA. By incorporating both physical and psychosocial domains, it offers a comprehensive evaluation that is well-suited for both clinical practice and research applications.

## 1 Introduction

Knee osteoarthritis (KOA) is a prevalent degenerative disease that commonly affects elderly populations. It is characterized by chronic pain, functional impairment, and significant limitations in activities of daily living (1). Recent data from the China Health and Retirement Longitudinal Study indicate that the overall prevalence of symptomatic KOA is reported to be approximately 8.1% in people aged 45 and older (2, 3). For patients with end-stage KOA, total knee arthroplasty (TKA) is widely regarded as an effective surgical intervention to alleviate symptoms and enhance knee function (2). Nonetheless, despite TKA has demonstrated clear benefits in reducing pain and improving mobility, a substantial subset of patients remains dissatisfied post-surgery (4). Research has linked such dissatisfaction to unmet preoperative expectations, which play a critical determinant of postoperative satisfaction (5–7).

Addressing this issue requires a broader view of patient needs beyond pain control and functional recovery. Many patients undergoing TKA aspire to regain higher levels of physical activity and participation in daily tasks, which are essential to their overall quality of life (8–10). Consequently, the evaluation of surgical outcomes must extend beyond traditional clinical metrics to incorporate patient-reported outcomes (PROs). These measures offer valuable information regarding patients’ own perceptions of recovery, especially regarding their ability to engage in meaningful daily activities (11, 12). Effective tools are crucial for comprehensively understanding the impact of TKA on patients’ lives and for guiding tailored interventions to meet their expectations.

Developed by researchers in Japan, the 25-question Geriatric Locomotive Function Scale (GLFS-25) is a self-administered tool designed to evaluate the locomotor impairment in elderly individuals, particularly those with age-related musculoskeletal conditions such as KOA (13). The scale consists of 25 questions that assess a variety of daily life factors, including mobility, pain, and psychosocial well-being (13). What sets the GLFS-25 apart is its comprehensive, multidimensional approach to locomotor function. Unlike other assessments that focus primarily on physical limitations, this scale also includes psychological and social impacts, such as emotional distress, mental health, and social engagement (14). The GLFS-25 provides a more extensive understanding of KOA’s effect on individuals’ lives. Though it has demonstrated strong reliability, validity, and sensitivity in the English version (13), it has largely remained confined to use in Japan and English-speaking regions.

In China, KOA poses a significant public health concern, especially among older populations, given its high prevalence and profound impact on daily activities and overall wellbeing (15). With the growing demand for TKA in patients with end-stage KOA, it is critical to have reliable tools to assess PROs. Although widely used tools like the Western Ontario and McMaster Universities Osteoarthritis Index (WOMAC) and Oxford Knee Score (OKS) have been translated and validated for use in China, they tend to concentrate on physical limitations and pain rather than the psychosocial dimensions of mobility-related quality of life (16, 17). Conversely, the GLFS-25 offers a more holistic approach, encompassing both the physical and psychosocial impacts of KOA (18). Given its comprehensive evaluation method, validating the GLFS-25 in China would provide a more complete assessment of the disease’s effect on patients’ lives.

The purpose of the present study was to translate and cross-culturally adapt the GLFS-25 scale for use in mainland China, and to validate the Chinese version of the GLFS-25 (GLFS-25CV) as a reliable and practical measurement tool for evaluating postoperative outcomes in TKA patients. We conducted a comprehensive analysis of its reliability, validity, and floor and ceiling effects in TKA patients.

## 2 Materials and methods

The study was approved by the Medical Ethics Committee of Honghui Hospital Affiliated to Xi’an Jiaotong University (Approval No: 202409021), and all participants provided written informed consent.

### 2.1 Translation and cross-cultural adaptation

The process of translation and cross-cultural adaptation of the English GLFS-25 into Chinese version was conducted according to international guidelines (19), including the following steps:

*Step 1.* Forward translation. The original English version of the GLFS-25 was translated independently to Chinese by two native Chinese speakers, including one professional standard translator and one orthopedic surgeon specializing in the diagnosis and treatment of KOA. The inconsistencies regarding the two translated drafts were discussed with three other Chinese orthopedic surgeons to reach a consensus. The 1*^st^* GLFS-25 CV was produced.
*Step 2.* Backward translation. The 1*^st^* GLFS-25 CV was translated back into English by two native English speakers with knowledge of the Chinese language, including one expert in orthopedic and one without medical background. Both of them were blinded to the original English version of GLFS-25. The backward-translated drafts were compared with the resource version and check for discrepancies. Step 1 and 2 were continued until there was no disagreements between the English and Chinese versions.
*Step 3.* Consensus meeting. A consensus committee including a psychologist, an orthopedic surgeon, a nursing specialist, a physiotherapist and four translators reviewed all of the version of GLFS-25 and resolved inconsistencies. Once language equivalency was achieved, the pre-final GLFS-25CV was produced.
*Step 4.* Preliminary test. The pre-final GLFS-25CV was administrated to 30 patients with end-stage KOA for a pretest. After the pretest, all the patients were asked to describe any difficulties in understanding and answering the questionnaires. Further refinement of the questionnaire was conducted by consensus committee according to the pretest results, then the final GLFS-25CV was developed.

### 2.2 Participants

Patients with end-stage KOA who scheduled to undergo TKA were recruited from the department of knee joint surgery, Honghui hospital, Xi’an Jiaotong University. The inclusion criteria for this study were as follows: (a) patients with end-stage KOA who were scheduled to undergo a primary unilateral TKA; (b) whose cognitive level can meet the requirements of completing the questionnaire; (c) who were fluent in Mandarin at a conversational level, and (d) consent to participate. Exclusion criteria were: (a) patients had history of other vascular, nerve, muscle, and bone diseases that affect movement or produce pain symptoms, such as hemiplegia, fracture, ligament injury, lower extremity vascular injury, etc.; (b) patients with serious diseases that affect daily life, such as coronary heart disease, asthma, mental illness, etc.

All self-assessment questionnaires were administered by a trained interviewer who asked the participants to complete the questions by themselves after undergoing TKA. If participants had difficulty in understanding the questions, they could consult with the interviewer at any time. We estimated the required sample size for analysis as follows: assuming a null hypothesis intra-class correlation coefficients (ICC) of 0.70 and an alternative hypothesis ICC of 0.90, with α = 0.05, power = 0.90, and two repeated measures, we determined that a minimum of 25 participants would be sufficient (20). To evaluate test-retest reliability, 35 patients from the first interview were selected using a random number table and were asked to complete the GLFS-25CV again at a 1 week interval by a second interview before TKA.

### 2.3 Questionaries

Every participant was required to fill the general demographic information questionnaire, including age, sex, height and weight, body mass index (BMI), educational levels, employment status, living status (live alone, live with spouse, live with offspring, live with domestic helper), and duration of pain.

The Chinese version of OKS questionnaire consists of 12 questions and it is divided into separate OKS pain and function subscales (21). Each question has five Likert-type answers ranging from 0 “significant disability” to 4 “no problem”, a total score is then calculated ranges from 0 to 48, with lower scores corresponding to poor function and more pain (11). The Chinese version of OKS has shown good content and convergent validity, test-retest reliability, internal consistency among elderly patients with KOA (21).

The Chinese version of WOMAC is a 24-item, disease-specific, self-administrated scale, patients are asked to rate each item on a five-point Likert scale (16). The WOMAC questionnaire consists of three domains, including: stiffness (two items; range, 0–8), pain (five items; range, 0–20), physical function (17 items; range, 0–68) (22). The three domain scores are then summed to create a total score (range, 0–96), where higher scores represent more pain, stiffness, and poor function. The Chinese version of the WOMAC has been validated previously (16).

The Chinese version of five-Level EuroQol Five-Dimensional Questionnaire (EQ-5D-5L) is an improved version of the health status questionnaire used to assess health-related quality of life (23, 24). It includes five dimensions: mobility, self-care, usual activities, pain/discomfort, and anxiety/depression. Each dimension has five levels: no problems, slight problems, moderate problems, severe problems, and extreme problems/unable (25, 26). The five-level design offers more detail compared to the original three-level version defining a total of 3,125 health states, which allows for better differentiation of an individual’s health status and improves the accuracy of the assessment (27–29).

The 25-question Geriatric Locomotive Function Scale is a self-administered questionnaire developed to assess locomotive syndrome or musculoskeletal functional decline in older adults (13). It comprises 25 items covering pain, activities of daily living, social functions, and mental health status. Each item is scored on a five-point Likert-type scale, ranging from 0 (“no difficulty/problem”) to 4 (“severe difficulty/problem”), for a total possible score of 0 to 100. Higher scores indicate more severe functional impairment or greater risk of locomotive syndrome (13).

### 2.4 Sample size determination

The sample size was calculated based on the COSMIN guidelines for assessing EFA. We followed the recommendations for EFA outlined in the guidelines, which suggest a ratio of 4–10 participants per item, with a total sample size exceeding 100 participants (30). The final sample size for this study was determined to be ≥ 250 participants. This ensures sufficient power and robust estimation of correlation coefficients, reflecting the strength of the relationship between the GLFS-25CV and related scales.

### 2.5 Statistical analysis

Descriptive statistics were used to summarize the demographic and clinical characteristics of the participants. All statistical analyses were performed with R 4.4.1 for Windows. The level of significance was set to 0.05.

#### 2.5.1 Structural validity

Exploratory factor analysis methods were used to examine the structural validity of the GLFS-25CV in order to assess the number of factors. The adequacy of these analyses was determined through the Kaiser-Meyer-Olkin test and Bartlett’s Test of Sphericity. EFA was conducted using the Maximum Likelihood method with Oblimin rotation. To determine the number of factors to be extracted in the EFA, the Parallel Analysis method was employed. Parallel analysis helps identify significant factor structures in the data by comparing the eigenvalues of the actual data with those from simulated data. A loading cutoff value of 0.4 was applied (31).

#### 2.5.2 Convergent validity

The Pearson correlation coefficient was used to test the convergent validity of the GLFS-25CV with OKS, WOMAC and EQ-5D-5L. The correlation levels were set as follows: rho > 0.59, strong correlation; 0.40 ≤ rho ≤ 0.59, moderate correlation; 0.20 ≤ rho ≤ 0.39, weak correlation; and rho < 0.20, very weak correlation (30).

#### 2.5.3 Internal consistency, test-retest reliability and measurement error—standard error of measurement (SEM) and 95 % smallest detectable change (SDC_95_)

Standard deviations (SD) and Cronbach’s α coefficient was used to evaluate the internal consistency. The SD of the item score higher than 0.85 and all the Cronbach’s α coefficients were higher than 0.9 indicate each item correlated with the whole scale and the internal consistency was excellent (32). Test-retest reliability was examined using the ICC, and an ICC value over 0.70 was considered to be evidence of good test-retest reliability (32). Measurement error was further quantified by SEM and the SDC_95_:


$$\mathrm{SEM}=\mathrm{SD}\times \sqrt{\left(1-\mathrm{ICC}\right)},{\mathrm{SDC}}_{95}=1.96\times \sqrt{2}\times \mathrm{SEM}.$$

#### 2.5.4 Floor and ceiling effects

The occurrence of floor and ceiling effects were analyzed by calculating the proportions of scores. If more than 15% of the participant achieved the highest or lowest score, this is indicative of a ceiling/floor effect on the corresponding item (33).

#### 2.5.5 Feasibility

Every participant was asked about any difficulties encountered during the answering process. The responsiveness was evaluated according to the percentage of missing item, the percentage of participants that did not answer some item, as well as the completion rate and time required to complete the questionnaire.

## 3 Results

### 3.1 Cross-cultural adaptation

During the translation and cross-cultural adaptation process, all 25 items of the GLFS-25 were successfully translated and underwent a rigorous evaluation to ensure both linguistic and cultural equivalence. No items were discarded or deemed inappropriate for the Chinese cultural context. Minor semantic adjustments were made during the consensus meeting to enhance clarity and relevance. The preliminary testing phase demonstrated that the patients with end-stage KOA had no major difficulties in understanding or completing the questionnaire. Feedback from participants primarily focused on semantic adjustments to improve alignment with daily living experiences. These changes were incorporated into the final version to ensure cultural relevance and clarity. Ultimately, the finalized Chinese version, GLFS-25CV, preserved the original intent and psychometric integrity of the GLFS-25.

### 3.2 Participants

**FIGURE 1:**
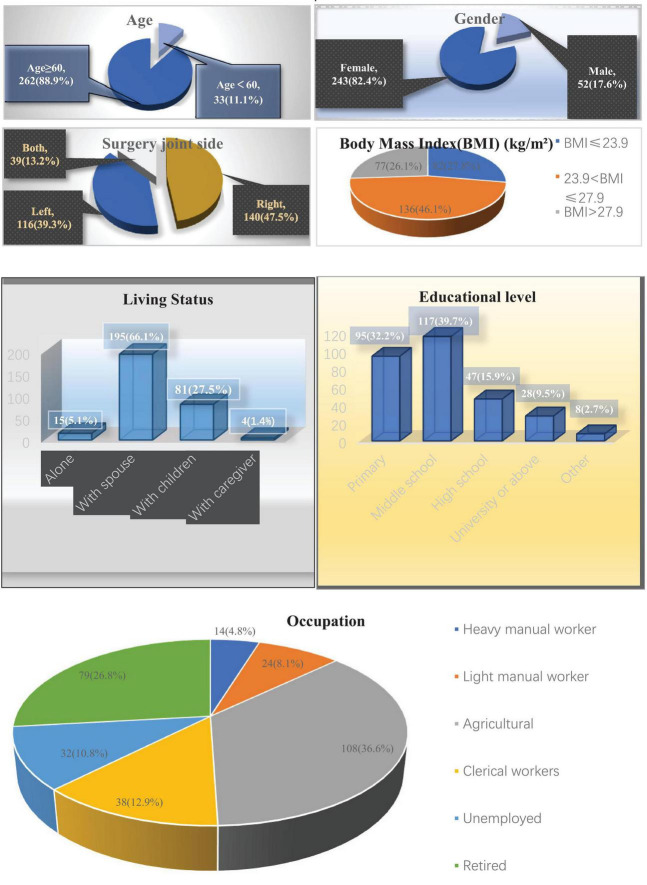
Patient characteristics (*n* = 295).

The demographic and clinical characteristics of the participants are shown in Figure 1. A total of 295 patients were enrolled. The cohort was predominantly elderly: 262 (88.9%) were ≥ 60 years old. Women made up 243 patients (82.4%) and men 52 (17.6%). Most procedures were unilateral—right knee 140 (47.5%) and left knee 116 (39.3 %)—with 39 individuals (13.2%) having bilateral surgery. According to Chinese cut-off values: BMI (kg/m^2^), 136 patients (46.1%) were obese (BMI > 27.9), 82 (27.8%) were overweight (23.9 < BMI ≤ 27.9), and 77 (26.1%) were in the normal-weight range (BMI ≤ 23.9).

### 3.3 Structural validity

**FIGURE 2:**
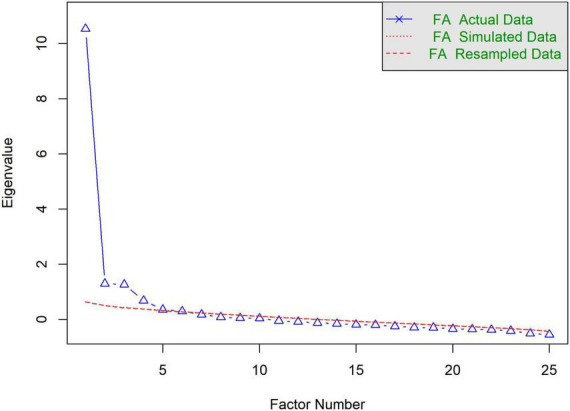
Parallel analysis scree plot.

**FIGURE 3:**
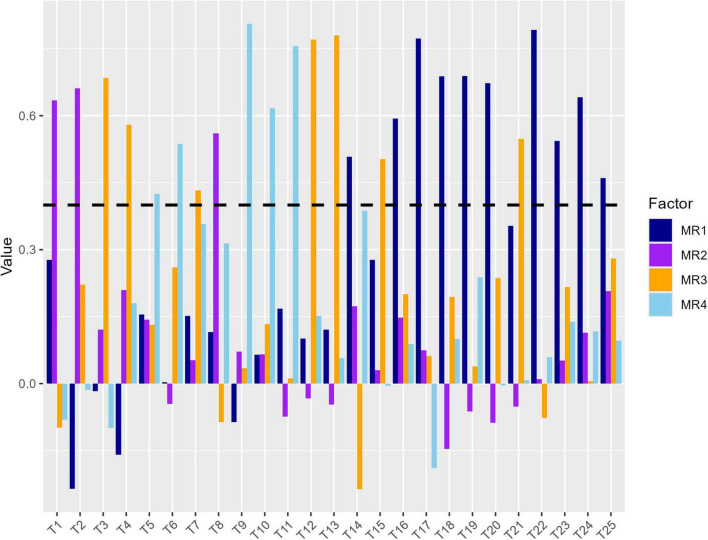
Factor loadings for Chinese version of the GLFS-25 (GLFS-25CV) items.

**FIGURE 4:**
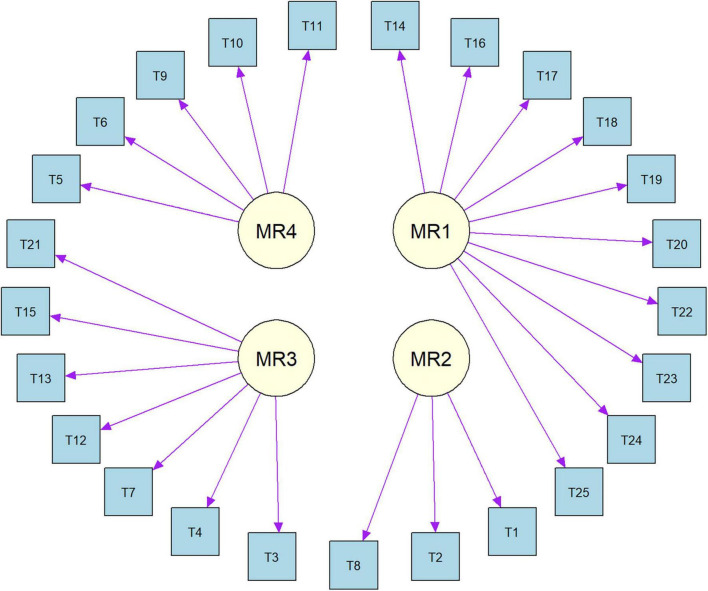
Factor structure path diagram for Chinese version of the GLFS-25 (GLFS-25CV).

The results of the Kaiser-Meyer-Olkin test (0.9) and Bartlett’s Test of Sphericity (*P* < 0.001) confirmed that the data are suitable for EFA. From the Parallel Analysis Scree Plot (Figure 2), it can be seen that the blue triangles represent the eigenvalues of the actual data, while the red dashed line represents the eigenvalues generated by simulation. Based on the plot, the number of factors with actual data eigenvalues exceeding the simulated data eigenvalues is four. Therefore, we selected four factors for further EFA. Figure 3 shows the factor loadings for the GLFS-25CV items, with factor loadings greater than 0.4 used as the threshold for classifying the items into factors. The variance explained by Factors 1–4 was 22%, 26%, 16%, and 14%, respectively. The cumulative variance explained was 58%, representing the total proportion of common variance accounted for by the four-factor model derived from the exploratory factor analysis. Figure 4 shows the path diagram depicting the relationships between the items and their corresponding factors. Items 14, 16, 17, 18, 19, 20, 22, 23, 24, and 25 loaded onto MR1, which was named “Motor Function Impairment.” Items 1, 2, and 8 loaded onto MR2, which was named “Upper Limb Dysfunction.” Items 3, 4, 7, 12, 13, 15, and 21 loaded onto MR3, which was named “Lower Limb Dysfunction.” Items 5, 6, 9, 10, and 11 loaded onto MR4, which was named “Activities of Daily Living Ability.”

### 3.4 Convergent validity

**FIGURE 5:**
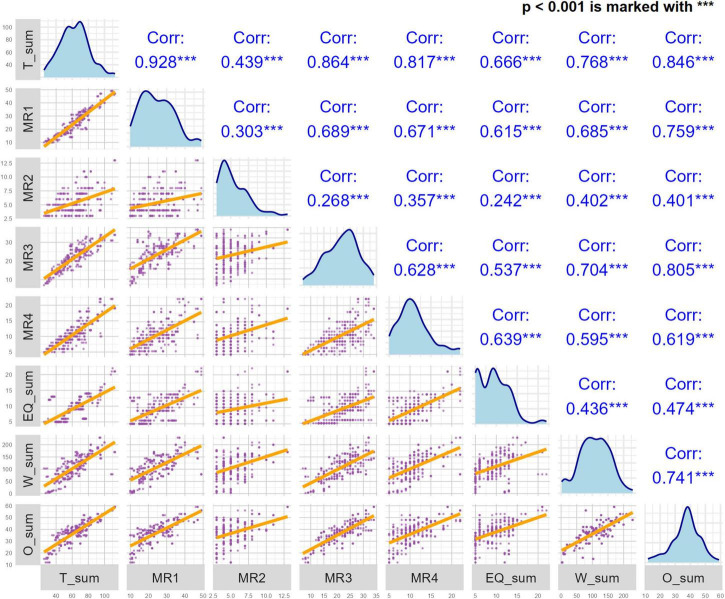
The correlations between the Chinese version of the GLFS-25 (GLFS-25CV) and Oxford Knee Score (OKS), Western Ontario and McMaster Universities Osteoarthritis Index (WOMAC), 5-Level EuroQol Five-Dimensional Questionnaire (EQ-5D-5L). EQ_sum, the total score for the EQ-5D-5L; W_sum, the total WOMAC score; O_sum, the total OKS score.

The correlations between the GLFS-25CV and OKS, WOMAC, EQ-5D-5L are shown in Figure 5. GLFS-25CV scores showed strong correlation with the total scores of the OKS (rho = 0.85), WOMAC (rho = 0.77), and EQ-5D-5L (rho = 0.66).

### 3.5 Internal consistency, test-retest reliability and measurement error (SEM and SDC_95_)

**FIGURE 6:**
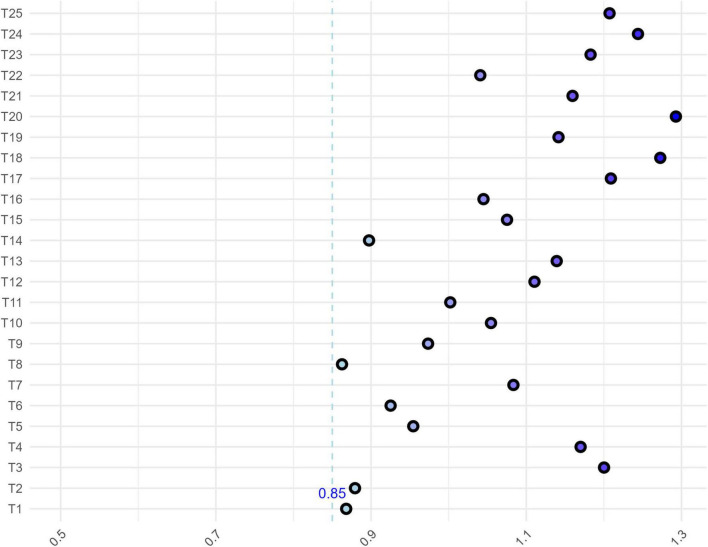
Standard deviations (SD) of Chinese version of the GLFS-25 (GLFS-25CV) items.

**FIGURE 7:**
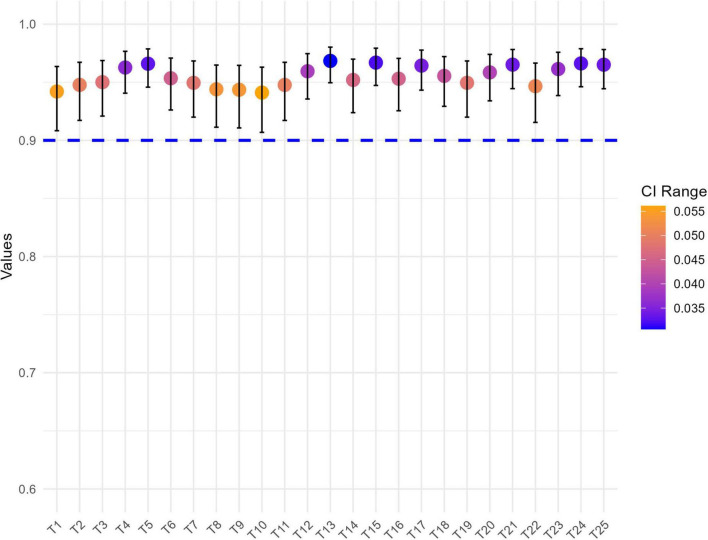
Test-retest reliability of Chinese version of the GLFS-25 (GLFS-25CV) with intra-class correlation coefficients (ICC) and 95% confidence intervals.

The GLFS-25CV exhibited excellent internal consistency, with Cronbach’s α value of 0.94 and all item SDs were higher than 0.85 (Figure 6) indicating that each item correlated with the whole scale. Test-retest reliability with a time interval of 1 week also showed good test-retest reliability, with an ICC ranging from 0.94 to 0.97 (Figure 7). Based on the GLFS-25 CV baseline total-score SD of 17.77, the SEM was 3.13, yielding an SDC_95_ of 8.67.

### 3.6 Floor and ceiling effects

For the summary score of the GLFS-25CV, both ceiling and floor effects were less than 1%, indicating a good distribution of the scale.

### 3.7 Feasibility

No difficulties were reported by participants during the completion of the questionnaire, and no missing or duplicate responses were identified. The completion rate of the GLFS-25CV was 100%, and the average time for completing the questionnaires was about 4.81 ± 0.51 min.

## 4 Discussion

The primary aim of this study was to translate and cross-culturally adapt the GLFS-25 for use in mainland China, and to validate the GLFS-25CV as a reliable and practical tool for evaluating postoperative outcomes in patients undergoing TKA for KOA. Our findings demonstrate that the GLFS-25CV is a robust and effective instrument for assessing knee function in Mainland Chinese populations, and the scale shows excellent validity and good reliability.

In terms of structural validity, the GLFS-25CV showed a clear, comprehensible factor structure, as evidenced by the EFA, which revealed a four-factor model. This finding aligns with previous research in diverse populations—including malignant tumor survivors with locomotive syndrome (31). These results support the cross-cultural applicability of the scale, as the identified factors are relevant to the Mainland Chinese populations, just as they were for other groups in earlier studies.

The convergent validity of GLFS-25CV was demonstrated, with its strong correlations with well-established instruments such as the WOMAC and OKS. Notably, correlations with OKS (rho = 0.85) and WOMAC (rho = 0.77), underscore that the GLFS-25CV measures functional outcomes in a manner aligned with these established tools. These results are consistent with those found in other linguistic adaptations of the GLFS-25, such as the Iranian version (rho = 0.86) (34) and the Brazilian version (rho ≥ 0.49) (14), further validating the scale’s cross-cultural convergent validity. These findings not only reinforce the effectiveness of the GLFS-25CV but also illustrates its potential for broader international deployment, particularly in cross-cultural studies on KOA and TKA outcomes.

The internal consistency of the GLFS-25CV was confirmed by Cronbach’s α reaching 0.94 and SD > 0.85, indicating excellent internal consistency, which aligns with findings from other national versions, including the Japanese (0.96) (13), Brazilian (0.94) (14), and Iranian (0.93) (34)versions, reflecting the internal consistency across diverse cultural and linguistic contexts. Additionally, the test-retest reliability of the GLFS-25CV was excellent, with ICC ranging from 0.94 to 0.97, which is higher than the ICC values reported for the Japanese version (0.71–0.92) (13), though slightly lower than the Brazilian version (0.976–0.984) (14). Nevertheless, all versions surpassed the 0.7 threshold, confirming good test-retest reliability in repeated measurements, and suggesting that the GLFS-25CV can consistently capture changes in knee function over time. This consistency underscores the GLFS-25CV’s suitability as a standard measure of locomotor function.

Feasibility was another pivotal aspect. Patients from varying educational backgrounds easily understood the GLFS-25CV and required an average completion time of just 4.81 ± 0.51 min. Such brevity and ease of use are critical advantages, particularly in clinical settings where time and patient compliance are paramount. The simple and clear design of the GLFS-25CV minimizes patient burden and improves compliance, making it a practical tool for widespread clinical use. Furthermore, these features are especially important in resource-limited settings or large-scale epidemiological studies, where patient adherence and efficiency are paramount. The ability to complete the scale quickly and easily enhances its practicality, providing clinicians with an effective tool for functional evaluation in KOA and TKA patients.

While the findings of this study are promising, several limitations should be considered. First, as participants were recruited from a single tertiary hospital in north-western China, the results may not reflect patterns seen in other regions; future multi-center work will be important for national confirmation. Second, because only postoperative data were gathered, the study could not evaluate responsiveness; longitudinal datasets incorporating both pre- and postoperative assessments are recommended. Third, women comprised the majority of the sample, limiting any sex-specific conclusions; larger, gender-balanced cohorts should be explored. Finally, qualitative follow-up in groups with lower literacy or distinct cultural backgrounds would help verify item comprehension and further fine-tune the GLFS-25CV for diverse settings.

In conclusion, the GLFS-25CV was successfully translated and culturally adapted, demonstrating excellent psychometric properties. The scale was found to be reliable, valid and easy to understand, making it a suitable tool for evaluating knee function in mainland China populations with KOA, post-operatively following TKA. The GLFS-25CV’s strong psychometric properties suggest it is a valuable instrument for clinical practice and research. We recommend its use in both clinical settings and large-scale research studies assessing the outcomes of TKA in China. Furthermore, additional studies are needed to explore the long-term responsiveness of the GLFS-25CV and its ability to capture changes in patient function over time, which would further establish its role in monitoring recovery in postoperative patients and potentially serve as a standardized instrument for international use in similar populations.

## Data Availability

The datasets used and analyzed during the current study are available from the corresponding authors on reasonable request.
